# Fast Registration of Point Cloud Based on Custom Semantic Extraction

**DOI:** 10.3390/s22197479

**Published:** 2022-10-02

**Authors:** Jianing Wu, Zhang Xiao, Fan Chen, Tianlin Peng, Zhi Xiong, Fengwei Yuan

**Affiliations:** 1 School of Mechanical Engineering, University of South China, Hengyang 421001, China; 2 School of Wealth Management, Ningbo University of Finance & Economics, Ningbo 315000, China; 3 School of Computer Science, University of Glasgow, Glasgow G12 8RZ, UK

**Keywords:** point cloud segmentation, 3D feature extraction, local features, local domain selection, regional semantic scoring

## Abstract

With the increase in the amount of 3D point cloud data and the wide application of point cloud registration in various fields, the question of whether it is possible to quickly extract the key points of registration and perform accurate coarse registration has become a question to be urgently answered. In this paper, we proposed a novel semantic segmentation algorithm that enables the extracted feature point cloud to have a clustering effect for fast registration. First of all, an adaptive technique was proposed to determine the domain radius of a local point. Secondly, the feature intensity of the point is scored through the regional fluctuation coefficient and stationary coefficient calculated by the normal vector, and the high feature region to be registered is preliminarily determined. In the end, FPFH is used to describe the geometric features of the extracted semantic feature point cloud, so as to realize the coarse registration from the local point cloud to the overall point cloud. The results show that the point cloud can be roughly segmented based on the uniqueness of semantic features. The use of a semantic feature point cloud can make the point cloud have a very fast response speed based on the accuracy of coarse registration, almost equal to that of using the original point cloud, which is conducive to the rapid determination of the initial attitude.

## 1. Introduction

With the development of 3D LIDAR [1,2], the 3D point cloud model has been widely spread in various fields, and engineering, medicine, and other fields increasingly rely on 3D point cloud information. With the continuous upgrading of 3D scanning equipment and technology, people can obtain low-cost and high-precision 3D object point clouds, and point clouds have gradually become the main data format by which to express the world. Point cloud registration plays a key role in 3D reconstruction, such as 3D model reconstruction [3,4], real-time 3D modeling [5,6], and 3D positioning applications such as UAV positioning [7,8] and mine positioning [9].

Point cloud registration mostly adopts the strategy of coarse registration first and then fine registration [10,11,12]. Among many point cloud registration algorithms, the iterative closest point (ICP) algorithm described by BESL and McKay [13] and Rusinkiewicz and Levoy [14] can obtain better registration accuracy, which is an important registration method in the precision registration algorithm. The iterative closest point (ICP) algorithm has been improved many times. Agamenoni et al. [15] improved ICP by using probabilistic data association in 2016, which can obtain better robustness. Yang et al. [16] proposed a method of directly processing range data in 2002, and registered continuous views with sufficient overlapping area to obtain accurate conversion between views. Ji et al. [17] proposed a row hybrid least square method for point cloud registration in 2017. Shuntao et al. [18] used the point pair with a smaller Euclidean distance as the point to be matched to improve the registration accuracy and convergence speed in 2018. Kamencay et al. [19] combined the scale-invariant feature transform (SIFT) function with the k-nearest neighbor (KNN) algorithm in 2019 to weight the iterative closest point (ICP) algorithm to reduce the error. Yang et al. [20] weighted the sampled structured data in 2019, improving the registration accuracy under the same level of downsampled data. It can be seen that ICP has high requirements for the initial position of the point cloud, and the inaccuracy of coarse registration may cause local minimum or non-convergence.

How to use the extracted feature points of 3D data for fast and effective registration is a challenging problem. However, there is no consensus on the definition of feature points. There are different feature extraction methods, and the registration schemes proposed for different feature extraction methods are also very different. Böhm and Becker [21] used feature points extracted by SIFT for label-free registration of point clouds in 2007. Barnea and Filin [22] used three-dimensional Euclidean distance to pair the extracted key points in 2008. Rusu et al. [23] obtained richer point features by analyzing the 16D local feature histogram of each point in the point cloud in 2008, and selected persistent feature points by counting the different distance measures between the histogram features of each point and the average histogram of the point cloud. Experiments show that the algorithm can deal with the noise of laser scanning well. Rusu et al. [24] greatly reduced the calculation time while retaining most of the identification ability of PFH by caching the previously calculated values and modifying the theoretical formula in 2009. Sipiran et al. [25] proposed the Harris operator to detect points of interest in 3D meshes in 2011. Li et al. [26] proposed an improved Harris’ algorithm in 2018, which uses gradient changes to identify feature points to eliminate pseudofeature points. Ye et al. [27] proposed a RANSAC algorithm in the same year to eliminate the wrong matching in registration. Kleppe al et al. [28] used conformal geometric algebra as a descriptor to extract feature points for feature registration in 2018. Xian et al. [29] proposed a sift operator in 2019 to reduce the impact of scale factors in a key point search. Lu et al. [30] proposed to use the key points selected by the mean value of domain curvature for point cloud registration in 2020. Experiments show that the algorithm has a faster calculation speed, higher registration accuracy, and better anti-noise performance. Ye et al. [31] proposed the meta-PU point cloud upsampling network in 2021. The results show that using this upsampling network can achieve significant performance gains for point cloud classification. Zhou et al. [32] proposed an objective point cloud quality index with structure-guided resampling (SGR) to automatically evaluate the perceptually visual quality of 3D dense point clouds in 2022. Experiments show that this method can realize the disentanglement of known information to a certain extent so that the key points can be sampled more uniformly.

Although the above methods can obtain the key feature points of the point cloud well, they have more or fewer problems with the speed of getting key points. When the point cloud data is huge, its computing speed will also be doubled, which is not conducive to the real-time processing of point cloud data, and the accuracy cannot reach the highest level within a limited number of iterations. Moreover, it can be seen from the above that the curvature estimation and normal of points are widely used in the feature extraction of points. Therefore, this paper uses local semantic scoring to screen out the high feature area composed of effective key feature points before extracting rich point feature information, so as to avoid the redundancy of calculation and achieve a fast response.

In contrast to the above methods, this paper introduces the fluctuation coefficient and stationary coefficient of local fields and proposes a key point extraction and coarse registration method based on the semantic scoring system. We conduct detailed experiments to compare our method with state-of-the-art methods. Experiments show that the proposed algorithm has better speed and accuracy on the basis of ensuring noise resistance.

After this introduction, the source of the point cloud data and the principle of the method will be described in detail in the second Section 2. In Section 3, the effectiveness of the algorithm is verified by experiments, and our findings are summarized in Section 4.

## 2. Methods

### 2.1. Semantic Features

We focus on calculating points in the field near a laser point. The set of point $P_{k}$ of point $P_{i}$ in field *R* is defined as $V_{P_{i}}^{r}$:

(1) $$P_{k}\in V_{P_{i}}^{r}⟺∥P_{i}-P_{k}∥\leq r.$$

#### 2.1.1. Normal Vector Calculation

The normal vector is one of the important features of the point cloud, which is widely used in various feature extraction algorithms such as PFH, FPFH, etc. Accurate normal vector estimation plays a key role in many point cloud algorithms. Principal components analysis (PCA) is a data analysis method, which is often used to calculate the normal vector and curvature. Here, we need to use the normal vector to score the local area of a point on the point cloud semantically. For any point $P_{i}={\left(x_{i},y_{i},z_{i}\right)}^{T}$ in the point cloud *P*, the covariance analysis is performed on the point $P_{ij}\in V_{P_{i}}^{k}$ in its *K* field, and the calculated covariance matrix $E_{i}$ is as follows:

(2) $$\;E_{i}=\frac{1}{k}\sum_{j=1}^{k}\left(P_{ij}-P_{io}\right){\left(P_{ij}-P_{io}\right)}^{T},\;E_{i}v_{i}=\lambda_{i}v_{i},$$

where $P_{io}$ is the barycenter of the point $P_{i}$ neighborhood point, *k* is the number of $P_{i}$ neighborhood points, and $v_{i}$ and $\lambda_{i}$ represent the eigenvectors of $E_{i}$ and the eigenvalues corresponding to the Eigen objects, respectively. Sort the feature $\lambda_{i}$ values so that they satisfy $\lambda_{i}^{\left(1\right)}\leq \lambda_{i}^{\left(2\right)}\leq \lambda_{i}^{\left(3\right)}$, and then the direction $v_{i}^{\left(1\right)}$ of the feature vector corresponding to $\lambda_{i}^{\left(1\right)}$ is the direction with the smallest variance in the *k*-neighborhood of $P_{i}$. Finally, the normal vector $\vec{n_{i}}$ of $P_{i}$ is obtained by uniting $v_{i}^{\left(1\right)}$.

#### 2.1.2. Adaptive Regional Scale

In the process of point cloud collection, different collection devices and the distance of the collection point will cause a certain difference in the overall density of the point cloud, and the density of different regions of the same point cloud is also different. In this paper, FPS is used to sample the point cloud as a whole, and the local point cloud density of the sampling point is calculated based on the minimum distance representation of spatial Euclidean distance, and the average point density $\mu_{p}$ of the overall point cloud is roughly estimated, where dis(p,q) represents point *p* and any point in the point cloud. The distance of a point *q*—the minimum distance between point *p* and other points—is represented by $D_{p}$, and $N_{fps}$ is the number of sampling points. We have

(3) $$\{\begin{array}{c} D_{p}=min\left(dis\left(p,q\right)\right),q=1,\;2,\;\ldots \;,N,p\neq q\\ \mu_{p}=\frac{\sum_{i=1}^{N_{fps}}D_{p_{i}}}{N_{fps}} \end{array}.$$

**Figure 1:**
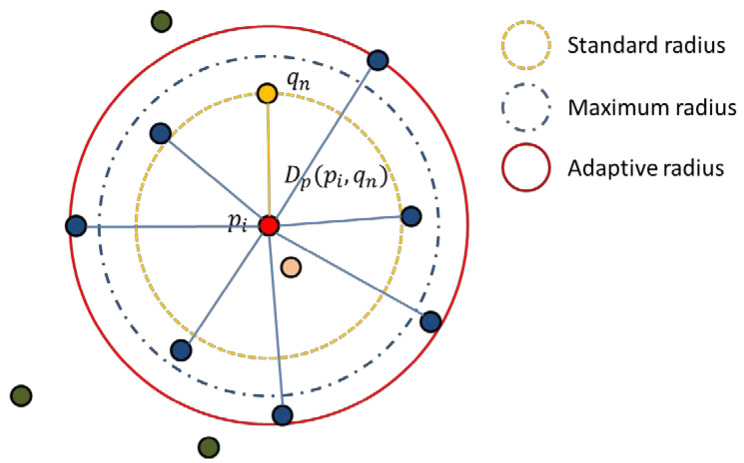
Schematic diagram of adaptive radius selection.

Here, we use the calculated $\mu_{p}$ to determine the adaptive area scale of the point cloud *p*, and to facilitate the selection of the Gaussian function bandwidth $\sigma^{2}$ below. The schematic diagram of the adaptive radius is shown in the following Figure 1. Selecting 2$\mu_{p}$ as the initial search radius of point *P* can effectively avoid the secondary query of most points to the field point. For all $q_{j}\in V_{P_{i}}^{2\mu_{p}}$, we search for the point $q_{m}$ and the next closest point $q_{n}$ with the Euclidean distance closest to $p_{i}$ in $V_{P_{i}}^{2\mu_{p}}$. The radius identification $S_{R}\left(i,m,n\right)$ is calculated according to Equation (4):

(4) $$S_{R}\left(i,m,n\right)=\frac{D_{p}\left(p_{i},q_{n}\right)}{D_{p}\left(p_{i},q_{m}\right)}.$$

If $S_{R}\left(i,m,n\right)<\beta$, and $D_{p}\left(p_{i},q_{n}{}_{m}\right)$ < 1.5$D_{p}\left(p_{i},q_{m}\right)$, taking $D_{p}\left(p_{i},q_{n}\right)$ as the standard radius of the point prefetching, we find the point cloud point in the specified range in $V_{P_{i}}^{2\mu_{p}}$ to determine the adaptive radius $R_{p_{i}}$ of the area, where $N_{rag}$ represents the range point that satisfies the condition, and then the adaptive radius $R_{p_{i}}$ is calculated by Equation (5):

(5) $$R_{p_{i}}=\frac{\sum_{j=1}^{N_{rag}}D_{p}\left(p_{i},q_{j}\right)}{N_{rag}},D_{p}\left(p_{i},q_{n}\right)<D_{p}\left(p_{i},q_{j}\right)<1.5D_{p}\left(p_{i},q_{n}\right).$$

When the points in the field do not meet the calculation requirements, the *K*-nearest neighbor search is used to replace the field search with a radius of 2$\mu_{p}$, but this method will cause the second repeated search of the area points and reduce the running speed.

#### 2.1.3. Semantic Scoring and Classification

In order to obtain the key points of the point cloud faster, the semantic score is used to classify the points. Compared with the simple rude method of using the surface undulation degree, by using Gaussian curvature or average curvature to obtain key points, this algorithm not only has an advantage in speed, but also semantically segments the point cloud, which facilitates the search for each key point, and can be better integrated into the subsequent algorithms and operations, bringing convenience to the processing of the point cloud.

By using the angle as a parameter to measure the fluctuation coefficient, the points in the local area of the semantic segmentation point are classified into the point set that needs to be scored later, and the average value of the included angle is obtained as the identification of this point:

(6) $$\{\begin{array}{c} \theta_{i}={cos}^{-1}\left(\frac{\vec{n_{i}}\cdot \vec{n_{j}}}{\left|\vec{n_{i}}\right|\times \left|\vec{n_{j}}\right|}\right)\\ \bar{\theta }=\frac{\sum_{i=0}^{n-1}\theta_{i}}{n} \end{array}.$$

In the formula, $\theta_{i}$ represents the angle between the normal vector $\vec{n_{i}}$ of the sampling point $P_{i}$ and the normal vector $\vec{n_{j}}$ of a point $P_{ij}$ in the *k* field. The larger the point, the greater the fluctuation of the area. We select the appropriate threshold $\delta_{\theta }$ to divide the points in the field into fluctuation points. We set $V_{P_{i_{m}}}^{r}$ and stationary point set $V_{P_{i_{n}}}^{r}$.

We use the following formulas to obtain the scores of the two point sets:

(7) $$Sr_{V_{P_{i_{d}}}^{r}}=\underset{P_{j}\in V_{P_{i_{d}}}^{r}}{\sum }w_{ij},\;w_{ij}=a\cdot exp\left\{-\frac{∥p_{i}-p_{j}^{r}∥{}^{2}}{\left(2\cdot \sigma_{scor}{}^{2}\right)}\right\}.$$

$w_{ij}$ is the Gaussian weight corresponding to the *j*th point in the field point set of the *i*th sampling point, where *a* represents the peak value of the Gaussian function, and its value determines the upper limit of the weight, which is taken as 1 here, $∥p_{i}-p_{j}^{r}∥$ represents the space Euclidean distance between the two points, which is a variable that affects the weight distribution, and $\sigma_{scor}{}^{2}$ is the bandwidth, which determines the difference of point weights within the sampling point field. Considering the influence factors of the nearest neighbors, the bandwidth value is consistent with the local point density 2$D_{p}$ of the point cloud. The point score calculation weighted by the Gaussian function of the field points improves the influence of the nearest neighbors on the score, reduces the interference of the far points on the score estimation, which fully takes into account the difference of the influence of the field points, and improves stability and noise immunity when the field radius is not properly selected.

Equation (8) is selected from the fluctuation coefficient $Sr_{p_{m}}$ and stationary coefficient $Sr_{p_{n}}$ of each point to describe the point degree ($Cg_{1}$), line degree ($Cg_{2}$), and surface degree($Cg_{3}$) within $V_{P_{i}}^{r}$:

(8) $$Cg_{1}=\frac{Sr_{p_{n}}}{Sr_{p_{m}}+Sr_{p_{n}}},\;Cg_{2}=\frac{Sr_{b}-kSr_{s}}{Sr_{p_{1}}+Sr_{p_{2}}},\;\;Cg_{3}=\frac{Sr_{p_{m}}}{Sr_{p_{m}}+Sr_{p_{n}}}.$$

Among these, $Sr_{b}=max\left(Sr_{p_{m}},Sr_{p_{n}}\right)$, $Sr_{s}$ represents the smaller coefficient of $Sr_{p_{m}}$ and $Sr_{p_{n}}$. The value of *K* is $\frac{1}{k_{1}}-1<k<\frac{1}{k_{1}}$, where $k_{1}$ is the ratio of $Sr_{s}$ and $Sr_{b}$, which is limited by the tolerance $\sigma_{Tolerance}$, and determines the boundary between the point degree ($Cg_{1}$), the line degree ($Cg_{2}$), and the surface degree ($Cg_{3}$). Here, the tolerance is generally set to $0.1<\sigma_{Tolerance}<0.2$, when $k_{1}<\sigma$, the value of *k* is $k<\frac{1}{k_{1}}-1$. Finally, the labels (1, 2, 3) in $V_{P_{i}}^{r}$ are defined by Equation (9):

(9) $$D^{*}\left(V_{P_{i}}^{r}\right)=arg\;\underset{d\in \left[1,3\right]}{min}\left[Cg_{d}\right].$$

If $Sr_{b}⋍kSr_{s}$, $Cg_{1}$ will be less than the other two, the result of the $D^{*}\left(V_{P_{i}}^{r}\right)$ tag is 1. Secondly, when $Sr_{p_{m}}\gg Sr_{p_{n}}$, it behaves as a corner point. Conversely, $Sr_{p_{n}}\gg Sr_{p_{m}}$ stands for $D^{*}\left(V_{P_{i}}^{r}\right)=$3.

In order to facilitate the selection of subsequent feature points, the points of all point clouds are classified according to labels, and the point set $P_{i}$ of $D^{*}\left(V_{P_{i}}^{k}\right)=$*d* is defined as $\cup_{d}$:

(10) $$P_{i}\in \cup_{d}⟺D^{*}\left(V_{P_{i}}^{r}\right)=d.$$

When the priority of feature point selection is $\cup_{1}>\cup_{2}>\cup_{3}$, use the above calculation to identify each point $\bar{\theta_{i}}$ and the maximum value $\theta_{max}$ in the set $\cup_{d}$, set the judgment threshold *T*, if $\bar{\bar{\theta_{i}}}>T$, then mark $P_{i}$ as the point cloud feature point set, where *T* and the relationship between the number of selected key points *N* is roughly judged, as shown in Equation (11):

(11) $$N=\frac{Size\left(\cup_{d}\right)}{\sqrt{2\pi }\sigma }\int_{T}^{\theta_{max}}e^{-\frac{{\left(x-\mu \right)}^{2}}{2\sigma^{2}}},\;N\leq Size\left(\cup_{d}\right).$$

Here, μ and σ represent the average value and variance of the $\cup_{d}$ midpoint angle identifier, and $Size\left(\cup_{d}\right)$ represents the number of $\cup_{d}$ midpoints. In order to obtain the threshold *T* according to the required *N* more quickly, the original formula is changed to $f\left(T\right)=\frac{Size\left(\cup_{d}\right)}{\sqrt{2\pi }\sigma }\int_{T}^{\theta_{max}}e^{-\frac{{\left(x-\mu \right)}^{2}}{2\sigma^{2}}}-N$, and the nonlinear Taylor expansion of Equation f(T) is as follows:

(12) $$f\left(T\right)=f\left(T_{0}\right)+f^{\prime }\left(T_{0}\right)\left(T-T_{0}\right)+\frac{f^{\prime \prime }\left(T_{0}\right){\left(T-T_{0}\right)}^{2}}{2!}+\ldots +\frac{f^{\left(n\right)}\left(T_{0}\right){\left(T-T_{0}\right)}^{n}}{n!}+R_{n}\left(x\right).$$

We take the first two terms as the linear part of the function, set it to 0 to get $f\left(T_{0}\right)+f^{\prime }\left(T_{0}\right)\left(T-T_{0}\right)=0$ and we use it as the approximate equation of the nonlinear equation f(T)=0, and obtain the iterative relationship as Equation (13), which helps to converge faster to A suitable threshold T:

(13) $$T_{n+1}=T_{n}-\frac{f\left(T_{n}\right)}{f^{\prime }\left(T_{n}\right)}.$$

When $N>Size\left(\cup_{d}\right)$, the excess part is searched in the next feature set, which achieves more precise quantity control than finding the threshold of a certain interval.

### 2.2. Coarse Registration Algorithm Based on Custom Semantic Feature Extraction

**Figure 2:**
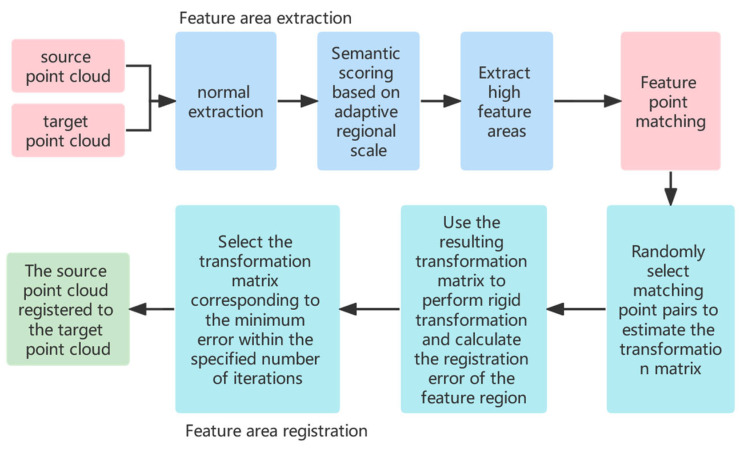
Point cloud registration method based on custom semantic feature extraction.

When performing high feature point registration, the high feature point sets from the source point cloud and the target point cloud are recorded as $P_{d}=\left\{p_{i}|p_{i}\in P,i=1,\;2,\ldots \;N\right\},Q_{d}=\left\{q_{j}|q_{j}\in Q,j=1,\;2,\;\ldots M\right\}$, where *P* and *Q* represent the source point cloud set and target point cloud set, respectively, and *N* and *M* represent the number of two high feature point sets, respectively. For any high feature point, this paper defines two metrics, namely the point feature similarity and the point-to-point feature similarity between the source point cloud and the target point cloud, and will satisfy a pair of two metrics at the same time. High feature points are regarded as a set of successful matching pairs. The flow chart of the registration method is shown in Figure 2.

#### 2.2.1. Feature Similarity between Points

For a high feature point $p_{i}$ in the source point cloud and any point $q_{j}$ in the target point cloud, its feature description vector is $p_{i\left(FPFH-\sigma \right)}=\left\{a_{1},a_{2},\ldots ,a_{34}\right\},q_{j\left(FPFH-\sigma \right)}=\left\{b_{1},b_{2},\ldots ,b_{34}\right\}$, of which the first 33 are FPFH features, and the 34th is the surface curvature calculated from the three eigenvalues $\lambda_{i}^{\left(1\right)},\;\lambda_{i}^{\left(2\right)},\;\lambda_{i}^{\left(3\right)}.$ Feature σ is denoted as Equation (14):

(14) $$\sigma =\frac{\lambda_{i}^{\left(3\right)}}{\lambda_{i}^{\left(1\right)}+\lambda_{i}^{\left(2\right)}+\lambda_{i}^{\left(3\right)}}.$$

If there are too many feature points extracted by custom semantics because the environment is too monotonous, we use the feature description vector of the points to sort the two point sets, filter out the points with higher characteristics, and then determine the point pair.

The Euclidean distance of its features is expressed as

(15) $$D_{FPFH-\sigma }\left(p_{i},q_{j}\right)=\sqrt{\overset{34}{\underset{i=1}{\sum }}{\left(a_{i}-b_{i}\right)}^{2}}.$$

If $D_{FPFH-\sigma }\left(p_{i},q_{j}\right)<\varepsilon_{\mathrm{Feature}}$, then we determine $p_{i}$ and $q_{j}$ as candidate corresponding points, find $n_{\mathrm{Feature}}$ $q_{j}$ that minimize $D_{FPFH-\sigma }\left(p_{i},q_{j}\right)$ in $Q_{d}$, and add point pair $\left(p_{i},q_{j}\right)$ to the corresponding candidate point set $C_{1}$. After this step is completed, the high feature point is completed. The initial matching between the feature points is followed by the matching between the feature point pairs.

**Figure 3:**
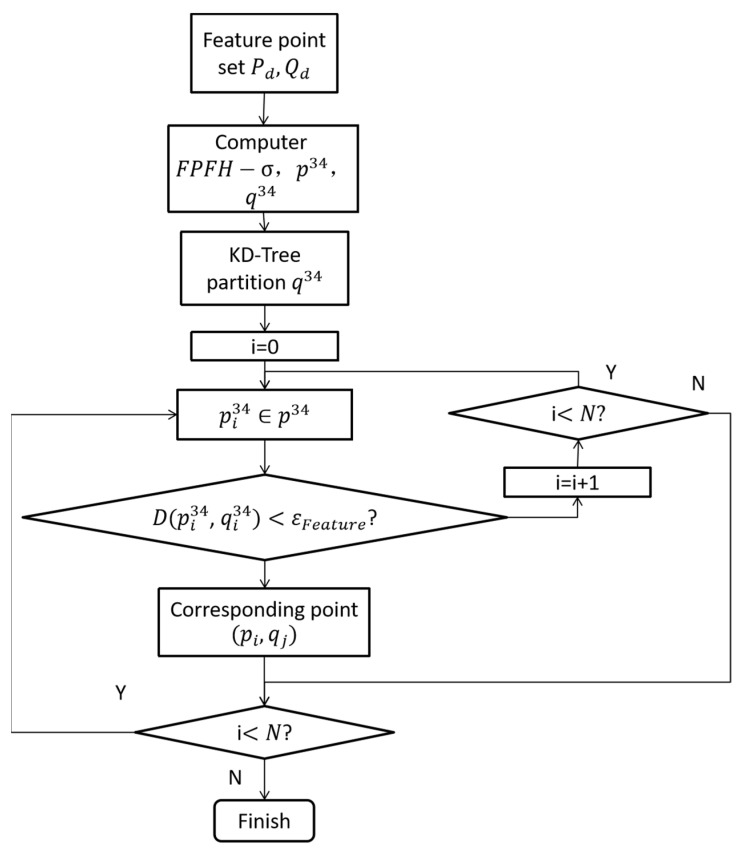
Feature matching process based on FPFH−σ.

In order to avoid traversing all feature points every time during the screening process, this paper uses *K*-dimensional tree (KD-Tree) [31] to search the range of *K*-dimensional data and the nearest neighbors, which has the characteristics of fast speed. The feature vectors of all the feature points of the cloud are, respectively, used as new dimension feature point clouds $p^{34},q^{34}$, and $q^{34}$, which are divided by KD-Tree to speed up the search for nearby points. Therefore, the flow chart of the feature matching is shown in Figure 3.

#### 2.2.2. Feature Similarity between Point Pairs

The candidate point set $C_{1}$ is denoted as $C_{1}=\left\{{\left(p_{i},q_{j}\right)}^{\left(k\right)},p_{i}\in P_{d},q_{j}\in Q_{d},k=1,\;2,\ldots N_{3}\right\}$. For any $p_{i}{}^{\left(k_{1}\right)},p_{i}{}^{\left(k_{2}\right)}\in P_{d}$, the distance between two adjacent points in the source point cloud is $d=∥p_{i}{}^{\left(k_{1}\right)}-p_{i}{}^{\left(k_{2}\right)}∥$.

The corresponding point found in the target point cloud should satisfy Equation (16):

(16) $$q_{j}\in Q_{d}\cap \left\{y|∥d^{\prime }-d∥<\varepsilon_{PartDis}\right\},$$

where: $d^{\prime }=∥q_{j}{}^{\left(k_{1}\right)}-q_{j}{}^{\left(k_{2}\right)}∥$, the search range of $q_{j}$ displayed by the threshold $\varepsilon_{Distance}$.

Due to the overall invariance of the point cloud, the distance parameters d between the point pairs must satisfy the above relationship, the parameter distance difference $D\left(p_{i},q_{j}\right)=∥p_{i}-q_{j}∥$ between the two matching points should also be consistent and satisfy the following relationship, so that the candidate point set for secondary evaluation. Among them, $D^{\prime }\left(p_{i},q_{j}\right)$ represents the difference of another pair of matching points,

(17) $$\left|D\left(p_{i},q_{j}\right)-D^{\prime }\left(p_{i},q_{j}\right)\right|<\varepsilon_{MatchDis}.$$

Finally, the corresponding points that satisfy the above constraints will form the corresponding point set required for registration.

### 2.3. Point Cloud Coarse Registration

Coarse registration estimates the rotation and translation matrix of the whole point cloud based on the correct matching points selected from the corresponding point set so that the rigid body of the source point cloud set changes to the coordinate system of the target point cloud. Considering the influence of the error on the matching point pair, this paper adopts SAC_IA to perform rough matching to increase the robustness of errors. The process is as follows:

1. Randomly select three points from the source feature cloud $P_{d}$, and obtain three sets of corresponding points for calculating the rotation and translation matrix V under the condition that the above constraints are satisfied.
2. Use the matrix V to perform rigid body transformation on the source high-feature point cloud sample set $P_{d}$, and the obtained sample point cloud set is recorded as $P_{dt}$.
3. For all points in the $P_{dt}$ point set, find the corresponding nearest points in the $Q_{d}$ point set respectively. Calculate its Euclidean distance, and use it as the estimated deviation *E* after accumulation.
4. Repeat the above three steps until the specified accuracy or the highest number of cycles is reached, and the minimum deviation $E_{min}$ obtained in the cycle is obtained. At this time, the corresponding rotation and translation matrix $E_{min}$ is $V_{min}$.
5. By using $V_{min}$, a rigid body transformation on the source point cloud *S*, calculate the deviation $E_{final}$ from the target point cloud set *T*.

## 3. Results and Analysis

### 3.1. Datasets

**Figure 4:**
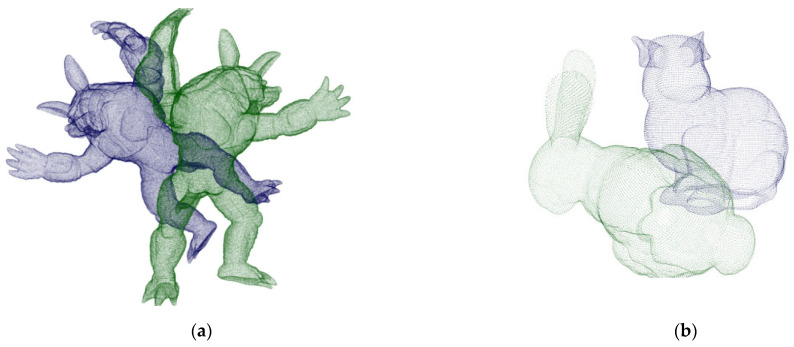
Initial point clouds. (**a**) Armadillo. (**b**) Bunny.

In order to verify the feasibility of the proposed algorithm, the standard models of “bunny” and “armadillo” in the 3D point cloud database of Stanford University are used for preliminary analysis. The address of the model is http://graphics.stanford.edu/data/3Dscanrep/ (accessed on 15 April 2022). The initial position of the point cloud is shown in Figure 4. Armadillo_ source, and bunny_ Source are the source point clouds represented in green. Armadillo_target, bunny_target is the transformed target point cloud represented by the blue point cloud.

**Figure 5:**
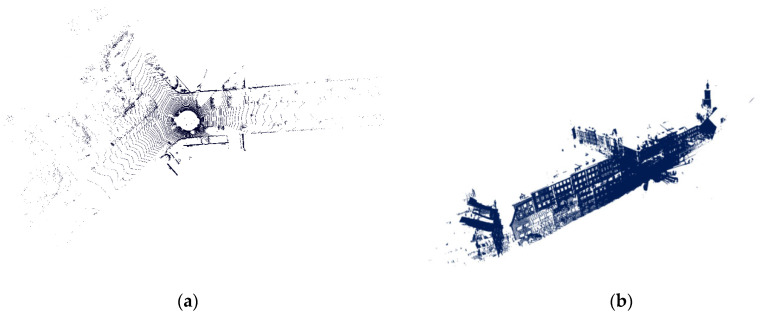
Outdoor point clouds. (**a**) KITTI Odometry Benchmark Velodyne point cloud. (**b**) Marketplace reduced.

After the preliminary experiment, in order to verify that the registration method proposed in this paper is also applicable to the registration of complex outdoor scenes, further evaluations were performed on the outdoor Semantic KITTI and Semantic3d datasets. In this paper, we used a reduced model named marketsquarefeldkirch4, shown in Figure 5b, which can be downloaded at http://www.semantic3d.net/ (accessed on 6 August 2022). Figure 5a shows the full 360-degree field of view of the employed automotive LIDAR collected while the vehicle is driving on the road, and this model is available at http://www.semantic-kitti.org/index.html (accessed on 13 September 2022).

### 3.2. Point Cloud Registration Results

#### 3.2.1. Generation Parameters Analysis

**Figure 6:**
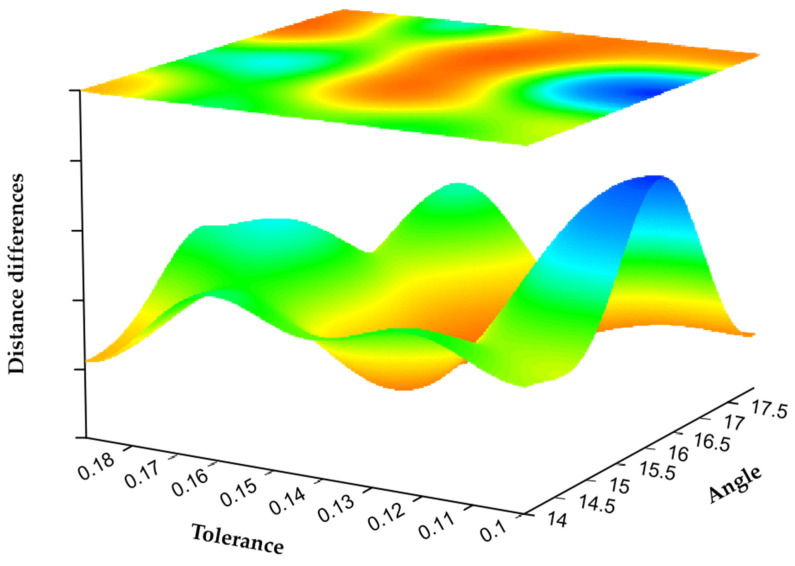
Registration error of different parameters.

The parameters required for semantic feature point extraction and point cloud registration of the two datasets are shown in Table 1. The parameter $n_{Feature}$ is the number of the required feature similarity between the corresponding points, which determines the accuracy of the registration points and the number of iterations. The parameter $\varepsilon_{MatchDis},\varepsilon_{PartDis}$ determines the accuracy of the secondary evaluation of the point pair, and the atmosphere represents the distance between the corresponding matching points of the source point cloud and the target point cloud, the threshold of the angle difference, and the distance threshold that needs to be reached between the corresponding point pairs. When these four parameters are small, higher matching accuracy can be obtained, but the operation rate will be reduced, and it may not be able to adapt to the registration situation with noise. In order to balance the influence of these three and obtain the best registration effect, we take $n_{Feature},\varepsilon_{MatchDis}$, $\varepsilon_{PartDis}$ as 3, 0.3$\mu_{p}$ and 4$D_{p}$. The parameter $\delta_{\theta }$ is the threshold that affects the fluctuation coefficient in the semantic scoring area, which controls the number of stationary points and fluctuation points, whereas the parameter $\sigma_{Tolerance}$ represents the use of the above point classification, which represents the boundary tolerance of feature point scoring. When the parameter $\delta_{\theta }$ is larger, the selection criteria of its feature points will be more stringent, which can reduce the number of feature points, but will blur its regional features. The parameter $\sigma_{scor}$ is used to reduce the interference of the far point on the scoring results. In order to have a better experimental effect, $\sigma_{scor}$ is taken as 17. The registration error adopts the nearest Euclidean distance, and the influence of different parameters on it is tested on the two datasets. In the experiment, we specified the range of $\sigma_{Tolerance}$ to be 0.1 to 0.2, with an interval of 0.01, the range of $\delta_{\theta }$ to be 14 to 18, with an interval of 1. In order to make the experimental results robust to noise, the two initial point clouds shown in Figure 4a were used, and all he points in armadillo_source are subjected to noise processing with a standard deviation of 1.25%$\mu_{p}$. The experimental results are shown in Figure 6. Surface fitting is performed by cosine series binary order 4 interpolation. This experiment shows that in a certain area (that is, $\sigma_{Tolerance}$) is in the range of 0.13 to 0.16 and $\delta_{\theta }$ is in the range of 14.5 to 17.5. The parameters have little effect on the algorithm, and the registration error is between 0.11 and 2.57. The error reaches a minimum value when the value is around (0.15,17). Therefore, in subsequent experiments, we specify the parameters $\sigma_{Tolerance}$ and $\delta_{\theta }$ as 0.15 and 17.

#### 3.2.2. Semantic Feature Point Extraction

**Figure 7:**
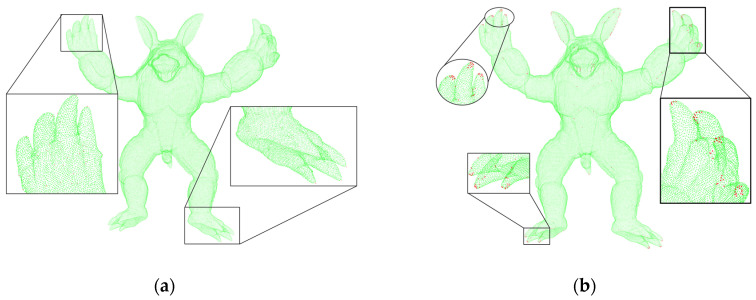
Feature point extraction. (**a**) 3D-Harris extraction. (**b**) Text semantic extraction.

As shown in Figure 7, the left side uses the 3D-Harris algorithm, the parameter is set to the normal vector estimation radius 1.5, and the key point estimates the feature corner points obtained by searching for the nearest neighbor radius 2, which are identified and distinguished by the red point cloud. On the right side are the key points obtained by the algorithm based on semantic scoring. It can be clearly seen that it has a good display of the area around the points with obvious features, which is conducive to the subsequent separate processing of the feature point cloud. By extracting its regional features, its running speed is significantly improved compared to the method of extracting the features of the whole point cloud.

**Figure 8:**
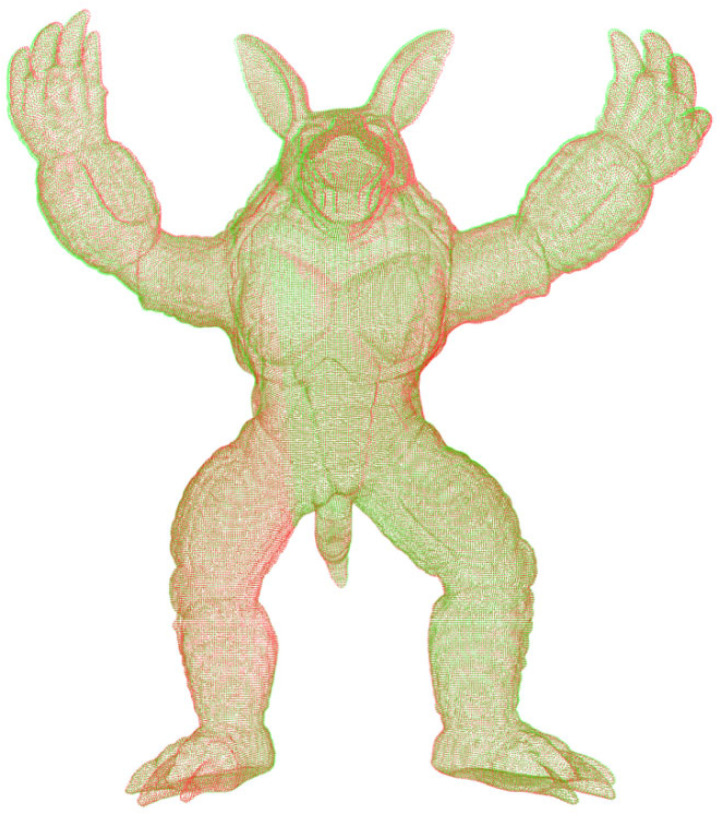
Point cloud register of armadillo.

For the initial point cloud shown in Figure 4a, this method is adopted, and the final registration map is shown in Figure 8.

### 3.3. Evaluation of the Proposed Method

#### 3.3.1. Time Performance

The registration experiments were carried out on a computer with a CPU Intel Core i5-5200U @2.2GHz, a hardware environment of 4G memory, and a software environment of the Windows 10 operating system, and code programming was performed in Visual Studio 2015 by using the C++ programming language and PCL library. Table 2 reflects the time required for each step of point cloud feature extraction and registration in the two datasets. From the time consumption table, we can see that the method has high time efficiency in registration, and can perform fast feature extraction and registration in the case of a large number of point clouds. The reason why this method has super high time efficiency for point cloud registration is that the simple and effective small-scale neighbor point collection is used to replace complex or large-scale feature extraction, and taking the method of extracting aggregated feature points instead of source point cloud to reduce the time cost of feature point extraction and registration.

#### 3.3.2. Comprehensive Analysis of Time Cost and Accuracy of the Proposed Method

The registration error is defined as the sum of the closest point distances between the point cloud to be registered and the target point cloud, and the time cost is defined as the time required to achieve the required registration error within the specified 10,000 iterations. We conducted ablation experiments to evaluate the impact of the custom semantic extraction and PFP_SAC proposed in this paper on the registration result. Table 3 presents method comparisons for the ablation study. The FPFH feature determines the persistent feature points and performs point cloud registration on them, which fully reflects the time consumption and registration accuracy of the original algorithm, which is convenient for comparing the advantages and disadvantages of the experimental results of the following new methods. The search radius of the FPFH of the method remains the same, and the number of feature points to be registered in the third, fourth, and fifth methods is the same. The 10 research results are averaged to obtain the comparison results shown in Table 4. Experiments show that the two parts of the method proposed in this paper are generally effective in independent experiments. When combined, the new method can obtain satisfactory registration results faster and can achieve better results when the number of point clouds is large. The reason that the semantic feature extraction has higher registration accuracy compared with other feature extractions is that the points extracted by the semantic features are distributed in its high feature area, and the resulting clustering effect is helpful for the subsequent point cloud registration.

#### 3.3.3. Registration Robustness Analysis

##### Robustness to Noise

**Figure 9:**
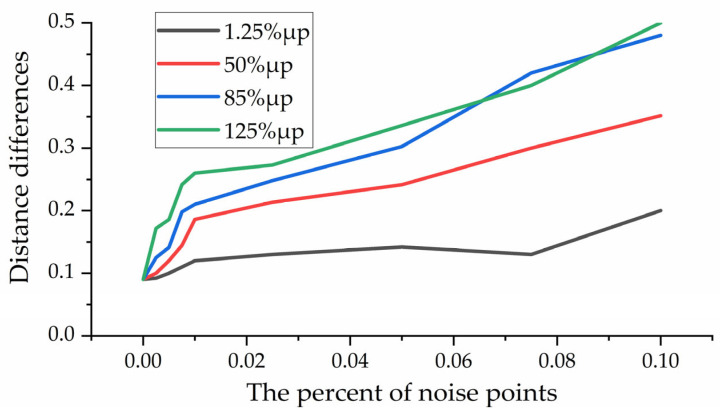
Noise robustness analysis.

To verify the robustness of the proposed method to noise, we added Gaussian noise with standard deviations of 1.25%, 50%, 85%, and 125% to the random number points in the Data A point cloud set, respectively. Figure 9 reflects the effect of different noises on registration accuracy. It can be seen from the figure that even under the influence of Gaussian noise as high as 1.25 times the point density, the method proposed in this paper can achieve high coarse registration accuracy. This experiment shows that the method in this paper has strong robustness to changing noise.

##### Robustness to Randomly Varying Point Density

**Figure 10:**
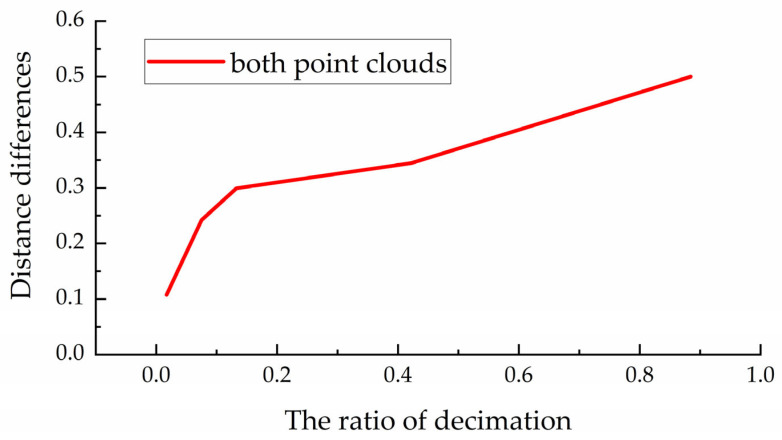
Robustness to varying point density.

In order to evaluate the influence of the variation of the point density caused by the pulse frequency or distance of the laser on the method proposed in this paper, the point cloud shown in Figure 4a was randomly downsampled to 1/18 of the original number of points, 9, 4/9, 8/9 to form point clouds with random density changes for verification. Figure 10 shows the effect of different point densities on the registration error. It can be seen that the proposed method still has good accuracy after randomly removing 8/9 points, which shows the robustness of the method in this paper to the randomly changing point density.

### 3.4. Outdoor Scene Application

**Figure 11:**
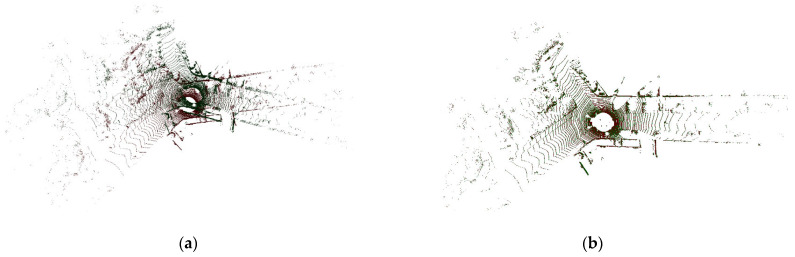
KITTI odometry benchmark velodyne point cloud. (**a**) Initial pose. (**b**) Registration rendering.

In order to verify that the method proposed in this paper is also suitable for high-challenging outdoor scenes, the point cloud image collected by the vehicle radar shown in Figure 5a is used for evaluation. Figure 11a is the initial pose map of the point cloud to be registered, and the registration result is shown in Figure 11b.

**Figure 12:**
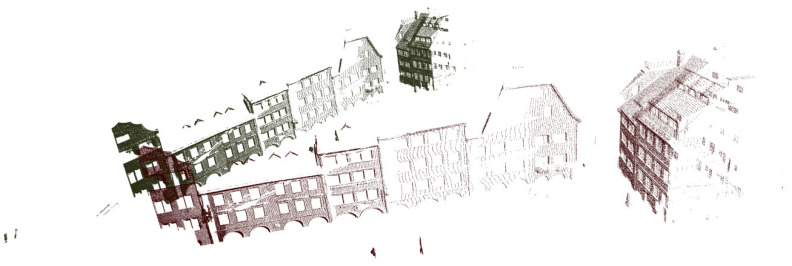
Partial outdoor point cloud registration initial position.

**Figure 13:**
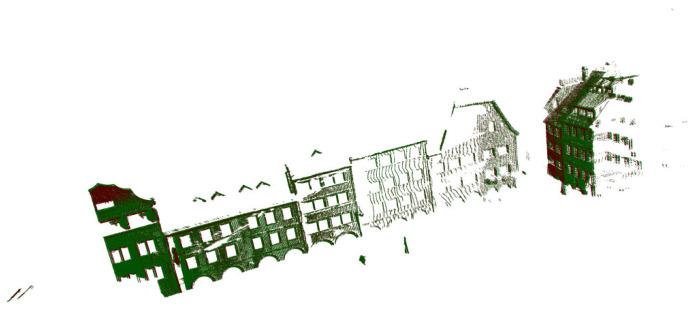
Optimal registration effect of some outdoor point clouds.

**Figure 14:**
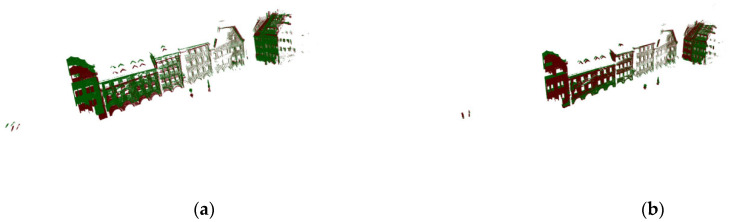
The registration effect corresponding to (**a**) 588 iterations and (**b**) 1412 iterations.

**Figure 15:**
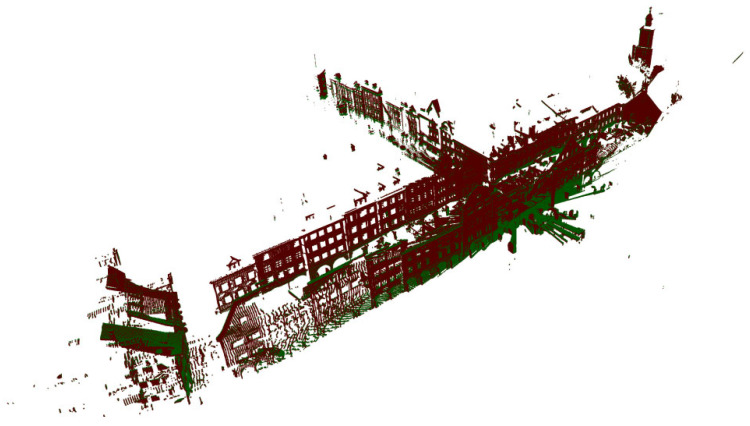
Registration of the whole point cloud.

Then we performed the method evaluation in urban point clouds and selected 172,974 points in the point cloud image shown in Figure 5b for preliminary simulation. After that, these points were appropriately rotated to obtain the initial image of the point cloud to be registered, as shown in Figure 12. The method proposed in this paper is used to register it. Due to the change in the model, we also slightly changed the parameter $\delta_{\theta }$ and set it to 19. As shown in Figure 13, when the number of iterations reaches 4251, the corresponding error was 0.06 Among them, Figure 14a,b corresponded to the renderings produced by 588 iterations and 1412 iterations, respectively. The previous experimental results did not further select high feature points. It can be seen that even when the features of the points are repeated many times, the method has good registration progress. After that, the overall point cloud is registered, and the final effect is shown in Figure 15.

Finally, we compared the method proposed in this paper with the classical P2P-ICP and P2L-ICP based registration methods. To reflect the impact of the number of high feature points on this method, we set the number of high feature points for the two scenes to 800 and 1500, respectively. The FPFH search radius is 0.5 and 0.3, respectively. The results are shown in Table 5. We can see that the method proposed in this paper still has a faster response speed on the basis of ensuring better registration accuracy in complex outdoor scenes, and the effect is most obvious in dense point clouds.

## 4. Conclusions

Fast coarse registration is a prerequisite for pose estimation, 3D scene reconstruction, and map localization. Aiming at the problems of slow registration of large-scale point clouds and a large amount of computation, a fast registration method of key regions based on semantic scoring is proposed. The important contribution of this paper lies in a new matching strategy that uses FPFH features for the registration of new feature point clouds formed by semantic feature points. Various experiments are conducted to evaluate the registration accuracy of the proposed method in various point cloud datasets and the robustness to different noise influences. Experiments show that the proposed method can have a faster running rate and higher registration accuracy under the premise of ensuring noise robustness, and can achieve a better matching effect for coarse registration. However, because FPFH is used as the feature of semantic feature points for matching, it does not necessarily have the best fit with this method, and further research is needed on the representation of point features. In addition, in-depth research on point clouds will be conducted in the future to study the remarkable effects that neural networks can produce in point clouds.

## Figures and Tables

**Table 1:** Parameter setting for experimental datasets.

Procedure		Parameter	Descriptor	Value
Semantic feature points extraction	Regional point cloud segmentation and scoring	$\delta_{\theta }$	Threshold for point set volatility coefficient	17
		$\sigma_{scor}$	Gaussian weight bandwidth in point set scoringata	2$D_{p}$
	High feature point extraction	$\sigma_{Tolerance}$	Tolerance of feature point extraction boundary	0.15
Point cloud registration	Correspondence matching	$n_{Feature}$	Feature similarity threshold for corresponding points	3
		$\varepsilon_{MatchDis}$	The distance threshold of the corresponding point	0.3$\mu_{p}$
		$\varepsilon_{PartDis}$	The maximum search distance of the corresponding point pair	4$D_{p}$

**Table 2:** Time performance of the proposed method.

	Semantic Feature Points Extraction (ms)		Point Cloud Registration		Total Time (s)
	Regional Point Cloud Segmentation	Feature Point Extraction	One Iteration (ms)	Number of Iterations	
Armadillo	3139	2034	1.2	1022	6.4
Bunny	536	358	0.2	94	0.9

**Table 3:** Comparison of methods for ablation studies.

	Persistent Feature Point Extraction	Point Feature Extraction	Registration
Method 1	FPFH	FPFH	SAC_IA
Method 2	Custom semantics	FPFH	SAC_IA
Method 3	FPFH	FPFH	PFP_SAC_IA
Method 4	Harris	FPFH	PFP_SAC_IA
Method 5	Custom semantics	FPFH	PFP_SAC_IA

**Table 4:** Performance comparison.

	Armadillo			Bunny		
	Registration Error	Time Cost (s)	Number of Iterations	Registration Error	Time Cost (s)	Number of Iterations
Method 1	0.068749	430.8	65	$1.77\times 10^{-15}$	10.67	28
Method 2	0.0989533	601.3	164	$2.192\times 10^{-13}$	3.71	94
Method 3	0.303254	56.9	10,000	$1.024\times 10^{-15}$	3.51	3552
Method 4	1.09559	24.5	10,000	$3.170\times 10^{-5}$	4.47	10,000
Method 5	0.0989533	6.4	164	$2.192\times 10^{-13}$	0.93	94

**Table 5:** Performance comparison in outdoor scenes.

	KITTI Odometry Benchmark Velodyne		Marketplace	
	Registration Error	Time Cost (s)	Registration Error	Time Cost (s)
P2P-ICP	0.00508164	27	23	291
P2L-ICP	0.00045351	21	103	276
4PCS	0.0791283	17	0.0415829	53
Our method	0.00492884	15	0.0253817	37

## Data Availability

Our code and dataset have been released at: https://github.com/Wujn1016/Semanti_Extraction (accessed on 22 August 2022).
